# Environmental and economic assessment of biochar production systems from agricultural residues

**DOI:** 10.1007/s42773-025-00527-2

**Published:** 2026-02-08

**Authors:** Yuzhou Tang, Judith Ford, Tim T. Cockerill

**Affiliations:** 1 School of Mechanical Engineering, University of Leeds, Leeds, LS2 9JT UK; 2 School of Geography, University of Leeds, Leeds, LS2 9JT UK

**Keywords:** Biochar, Farm-scale pyrolysis, Agricultural residues, Life cycle assessment, Techno-economic assessment, Greenhouse gas removal

## Abstract

The agricultural sector urgently requires scalable solutions to reduce greenhouse gas (GHG) emissions from residue management. Biochar offers a promising carbon removal pathway, but its adoption is limited by technical, regulatory, and economic barriers. A key constraint is the lack of system designs that can accommodate multiple feedstocks while complying with land application regulations. This study designs and evaluates an integrated biochar production system that enables the separate processing of straw and manure through parallel pyrolysis lines, while optimising internal energy use. Environmental and economic assessments were conducted using a case study of the University of Leeds Research Farm, under a cradle-to-grave system boundary. The results show that the system can produce 300 t of biochar annually, sequester 350 t CO_2_e, and reduce manure management emissions by 75%, with an additional 30 t CO_2_e avoided through surplus heat utilisation. The carbon abatement cost is estimated at £226 per t CO_2_e, primarily driven by capital (38%), operational (32%), and electricity (30%) costs. Sensitivity analysis highlights that straw availability, determined by both yield and crop rotation, is the primary factor influencing system performance. Among the mitigation strategies for addressing heat shortfalls, procuring external straw is identified as the most effective option. This study presents a novel and adaptable system framework for on-farm biochar deployment, addressing key barriers to implementation. The findings provide quantitative insights into the trade-offs between cost, carbon removal, and design decisions, and offer a foundation for scaling biochar use across the agricultural sector.

**Graphical Abstract:**

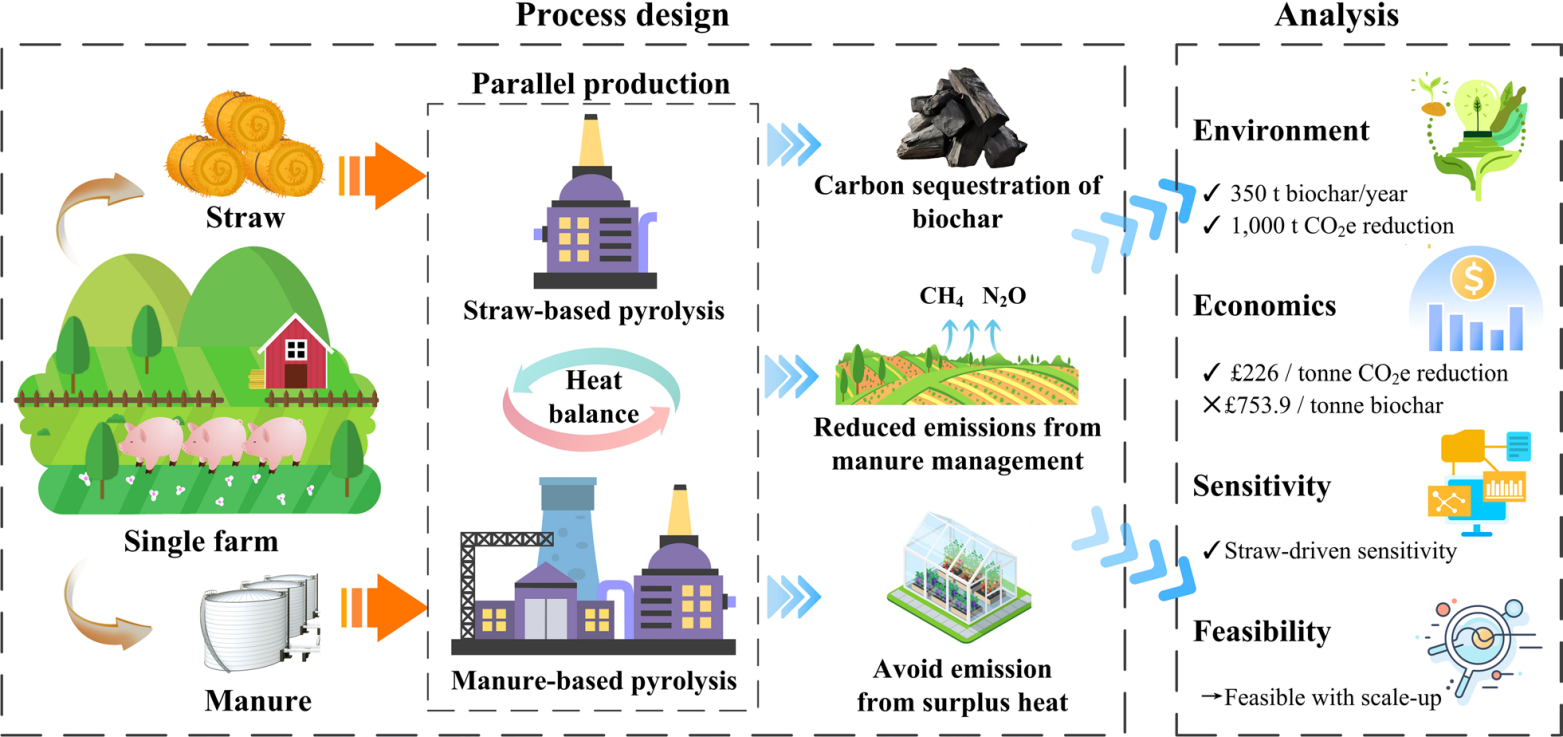

**Supplementary Information:**

The online version contains supplementary material available at 10.1007/s42773-025-00527-2.

## Highlights

- Parallel biochar system enables separate straw and manure processing while meeting regulatory requirements.
- Heat integration between straw and manure lines improves energy use and supports on-farm feasibility.
- Straw production variability affects system outcomes, highlighting the need for flexible operating strategies.

**Supplementary Information:**

The online version contains supplementary material available at 10.1007/s42773-025-00527-2.

## Introduction

As the United Kingdom (UK) progresses towards its 2050 net-zero target, the agricultural sector faces increasing pressure to reduce greenhouse gas (GHG) emissions. Agriculture contributes approximately 12% of the UK’s total GHG emissions (DESNZ 2024a), with manure management alone accounting for nearly 10% of emissions from the sector (Petersen et al. 2013). In parallel, the management of crop residues such as straw remains inefficient, often resulting in uncontrolled emissions or resource loss (Koul et al. 2022; Workman et al. 2022; Patel and Panwar 2023). This presents a significant opportunity to repurpose agricultural residues to support climate mitigation objectives.

Biochar represents an established engineered greenhouse gas removal (GGR) approach, involving the pyrolysis of biomass residues to produce a stable material suitable for long-term carbon sequestration (Chiquier et al. 2022). In addition to its carbon removal potential, biochar application can reduce soil GHG emissions and enhance soil functionality (Liu et al. 2023). Compared with technologies such as bioenergy with carbon capture and storage, biochar offers a more decentralised and potentially farm-based solution (Cueva Z et al. 2022; Hu et al. 2024; Castells et al. 2024; Jiang et al. 2024; Lee et al. 2024). Despite these advantages, the adoption of biochar systems within agriculture remains limited due to a combination of practical, technical, and regulatory barriers.

One major limitation is the lack of viable implementation models that are suitable for real-world farm settings. While a wide range of feedstocks such as straw and manure are readily available, they vary significantly in moisture content and processing requirements (Meng et al. 2021). Although co-pyrolysis of mixed feedstocks has been explored in some studies (Qi et al. 2024; Lian et al. 2023; Zhao et al. 2024), regulatory frameworks in both the UK and European Union currently prohibit the land application of biochar produced from mixed residues (EA 2019; EBC 2022). Moreover, the high moisture content of manure renders its processing particularly energy-intensive (Ro 2016). These challenges, coupled with the high capital and operational costs associated with small-scale systems, have restricted biochar adoption among farmers (Hu et al. 2024; Campion et al. 2023; Jaffé et al. 2013; Sakrabani et al. 2023).

Previous life cycle assessment (LCA) and techno-economic assessment (TEA) studies have highlighted the carbon sequestration potential of biochar(Hu et al. 2024; Zhu et al. 2022; Terlouw et al. 2021). However, few studies have proposed integrated biochar systems designs that are compliant with regulatory standards, enable feedstock separation, and improve energy efficiency under realistic operating conditions. There remains a pressing need to investigate whether a farm-based system can process multiple residue types in a regulation-compliant manner while delivering both environmental and economic benefits.

In this study, we introduced an integrated parallel biochar production model and evaluated it using data from the University of Leeds Research Farm. Unlike common configurations reported in the literature, the model processes straw and manure in dedicated lines to preserve regulatory compliance while enabling energy integration through reuse of surplus heat. This feedstock-specific design reduces the energy penalty associated with high-moisture manure, improves capacity utilisation under farm operating conditions, and retains operational flexibility as residue mixes vary over time. We coupled the process design with a farm-scale LCA and TEA to quantify climate benefits and costs. A sensitivity analysis was conducted to identify the key impact factors and to explore the strategic implications of these factors. Together, these innovations advance the state of the art by providing a farm-ready architecture that is both compliant and scalable, clarifying how biochar can contribute to on-farm decarbonisation.

## Methods

### Goal and scope

**Fig. 1:**
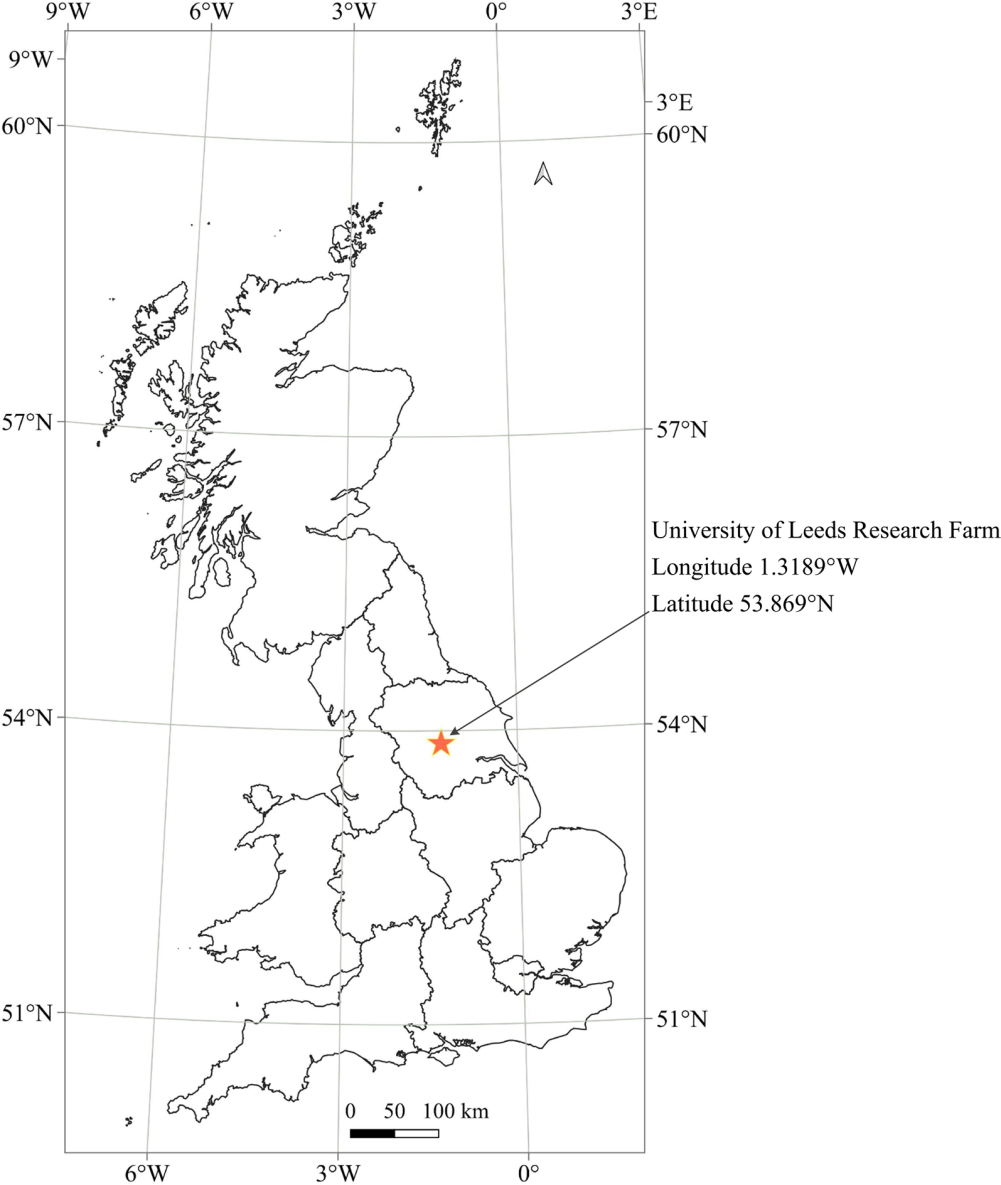
Location of the university of leeds research farm

The aim of this research was to propose and assess an integrated biochar production system for farm-scale application that enables the separate processing of multiple agricultural residues, aligns with regulatory requirements, and improves internal energy efficiency. Using a single representative small farm as a testbed, this study evaluated the technical feasibility and GHG removal potential of the proposed system, with the LCIA focused on climate change impacts. Broader environmental indicators and larger-scale applications will be addressed in future work. The system was evaluated using the University of Leeds Research Farm as a representative case study, under realistic operating conditions. The farm (UoL 2024) comprised 230 hectares of arable land managed through rotational cropping and supported over 6000 pigs (Fig. 1).

The main crops grown included winter wheat, winter barley, oilseed rape, peas, and potatoes. At present, pig manure is stored in tanks and applied to arable land during the spring and autumn. Approximately 400 metric tonnes (t) of straw are used annually for on-farm purposes. In the proposed biochar production system, straw and pig manure were processed through two parallel pyrolysis lines. Pig manure was separated into thick and thin fractions using a mechanical press, with the thick fraction used for biochar production and the thin fraction stored and subsequently spread on the land.

**Fig. 2:**
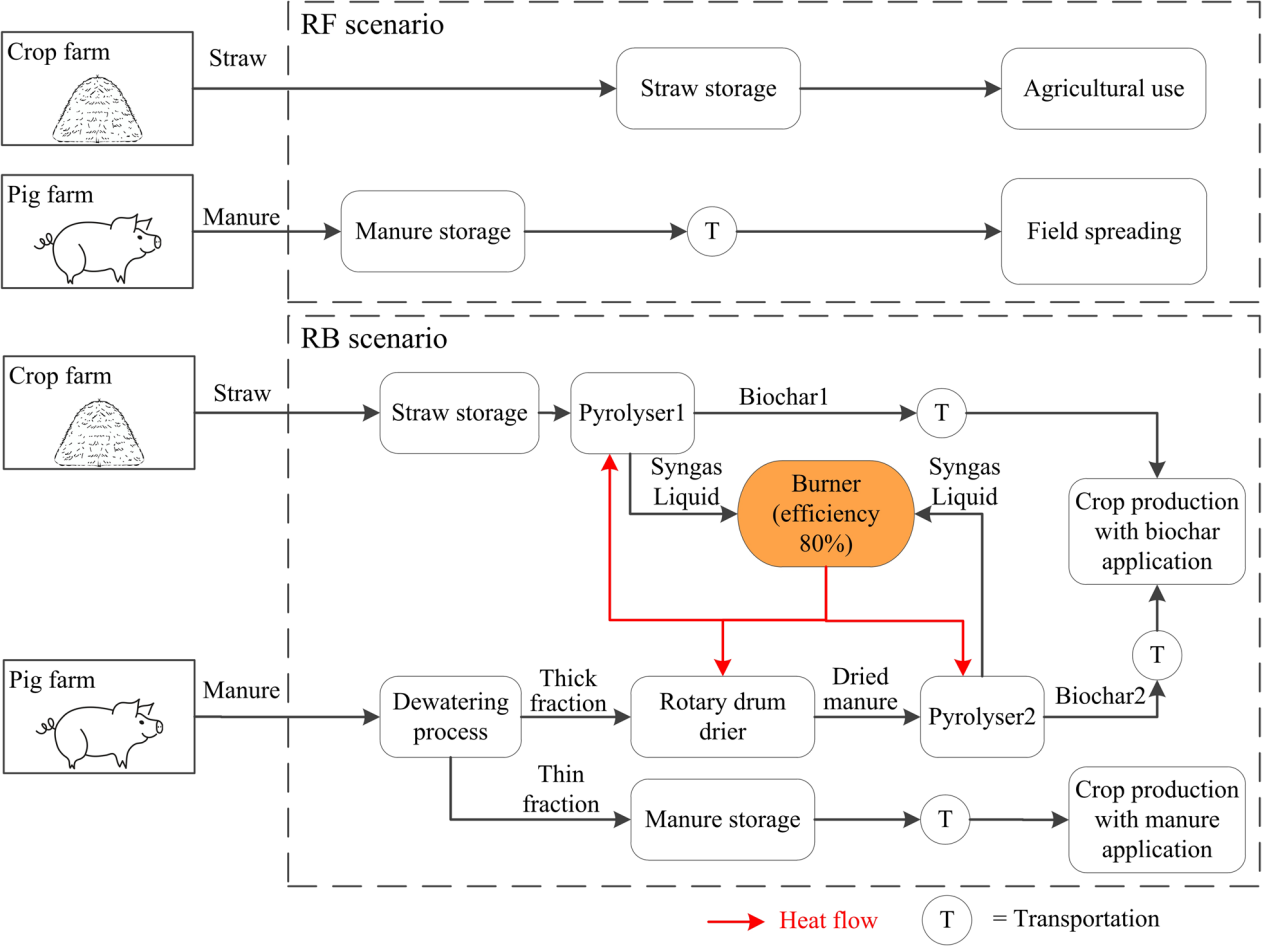
System boundary

The study adopted a one-year farm operation as the functional unit to compare the environmental impacts of the proposed biochar utilisation of agricultural residues (RB scenario) with those of the existing treatment practices (RF scenario). A cradle-to-grave approach was selected as the system boundary. To ensure consistency, the system boundary for both scenarios included the same quantities of straw and manure. For straw, the RF scenario modelled the surplus straw being used for agricultural purposes (e.g., animal bedding, soil incorporation), whereas in the RB scenario, the surplus straw was allocated to biochar production. Regarding manure, the RF scenario involved the direct field application of untreated manure, while in the RB scenario, manure was processed through a dewatering step, separating it into a thick and a thin fraction. The thick fraction was converted into biochar and applied to the field, while the thin fraction was managed similarly to the RF scenario through field application. The processes included within the system boundary are illustrated in Fig. 2. This study was based on system modelling and scenario analysis. Crop rotation data from 2021 to 2028 and sensitivity analyses were used to capture variability and assess the robustness of the results.

### Life cycle inventory

The life cycle inventory (LCI) for the baseline reference (RF) scenario primarily included GHG emissions from manure management. These data were sourced from the University of Leeds Farm GHG Inventory (2022–2023), which reports 889.4 t CO_2_e in annual scope 1 and 2 emissions. This inventory was conducted as part of the farm's environmental audit, and further details are available upon request.

The LCI for the biochar utilisation (RB) scenario was modelled over a one-year operational period using an hourly-resolution simulation of the biochar production system. The study was assumed to operate for 300 days annually on a continuous 24-h schedule, with the remaining days allocated to maintenance and inspection. Consistent with a cradle-to-grave system boundary, the RB scenario also accounted for potential emissions from any remaining untreated manure, which were assumed to be proportional to the dry matter (DM) content. A summary of the LCI for both scenarios is presented in Table 1. The following sections provide further detail of the RB scenario, including data assumptions and the energy modelling approach used.

**Table 1:** Life cycle inventory of two scenarios

Category		Item	Amount (Functional unit)	
			RF scenario	RB scenario
Straw biochar production	Input	Straw	330.1 t	330.1 t
Straw pyrolysis	Input	Electricity	/	117,184.9 kWh
		Water	/	193.5 m^3^
	Output	Straw-based biochar	/	87.4 t
Manure biochar production	Input	Farmyard manure	1750 t	1750 t
		Slurry	8000 t	8000 t
Press process	Input	Electricity	/	8223.6 kWh
Drying process	Input	Electricity	/	16,272.4 kWh
Manure pyrolysis	Input	Electricity	/	118,665.9 kWh
		Water	/	196.0 m^3^
Burner	Output	Manure-based biochar	/	208.1 t
	Output	Surplus heat	/	214.4 GJ
Manure application	Input	Transportation	8391.6 t·km	8035.9 t·km
	Output	Total CH_4_ CO_2_e	619.0 t	152.4 t
		Total N_2_O CO_2_e	270.4 t	66.6 t
Biochar spreading	Input	Transportation	/	295.5 t·km

#### Feedstock availability and characteristics

The RB scenario included two parallel pyrolysis lines that separately processed straw and the dewatered thick fraction of pig manure. According to 2023 farm records, the pig unit processed at full capacity, collecting 8000 m^3^ of slurry and 1750 t of farmyard manure (FYM). The DM content of the slurry was 4%, and the DM content of FYM was assumed to be 25% based on sample testing. Slurry is currently stored in four 1000 m^3^ tanks and applied to land.

Surplus straw from wheat, barley, and oilseed rape was allocated for biochar production. Cultivated areas and corresponding straw yields were based on 2023 farm data, except for oilseed rape straw yield, which was derived from the literature (Mathew et al. 2011), as shown in Table 2. Since the harvest period for these crops is concentrated between July and September, straw is collected in bales and stored for later use. It was assumed that the straw is naturally dried to a moisture content below 10% prior to storage, with an assumption of negligible degradation (Summers et al. 2003).

**Table 2:** Straw yield data of the farm

	Area (ha)	Straw yield (t/ha)	Straw amount (t)
Wheat	76.5	6.4	489.6
Winter barley	23.9	6.4	153.2
Oilseed rape	58.2	1.5	87.3
Sum			730.1

To ensure long-term carbon stability and compliance with certification standards, a pyrolysis temperature of 600 °C was adopted for both straw and manure feedstocks, as recommended by the European Biochar Certificate (EBC 2020). This also ensures the removal of biological hazards and micropollutants. Feedstock characteristics and pyrolysis properties, including the higher heating value (HHV) of both feedstocks and their resulting biochar, are detailed in Table 3.

**Table 3:** Characteristics and pyrolysis properties of feedstocks

	Barley straw^a^	Wheat straw^a^	Oilseed straw^b^	Indoor manure^c^	Slurry^c^
Feedstock characteristics					
Moisture of feedstock (%)	8.6	8.4	2.85	83	92.37
HHV of feedstock, dry basis, (MJ/kg)	17	17.1	12.04	14.8	14.8
Pyrolysis yield					
Biochar yield (%)	25.8	28.9	32.5	36.4	36.4
Liquid yield (%)	52.5	49.1	39.93	35	35
Syngas yield (%)	19.9	19.7	27.58	23.7	23.7
Biochar characteristics					
C (%)	67	67.2	67.85	43.9	43.9
Ash (%)	22.7	20.4	22.54	52.1	52.1
HHV (MJ/kg)	25.9	24.6	27.61	17	17
H:C molar ratio	0.36	0.36	0.30	0.41	0.41
N (%)	0.5	0.6	1.59	1.6	1.6
P (g kg^–1^)	2.1	2.1	2.9	20.34	20.34
K (g kg^–1^)	3.7	3.5	28.6	25.78	25.78
pH	10.40	10.20	10.41	12.54	12.54
Cd (mg kg^–1^)	bdl^d^	bdl	2.98	0.25	0.25
Pb (mg kg^–1^)	bdl	bdl	bdl	39.41	39.41
Hg (mg kg^–1^)	< 0.1	< 0.1	bdl	–	–
As (mg kg^–1^)	bdl	bdl	1.09	–	–
Cr (mg kg^–1^)	2	2	4.36	30.16	30.16
Ni (mg kg^–1^)	1.2	0.8	3.27	22.56	22.56
Cu (mg kg^–1^)	bdl	bdl	13.78	42.37	42.37
Zn (mg kg^–1^)	bdl	bdl	8.80	131.08	131.08

^a^Sedmihradská et al. 2020

^b^Qi et al. 2024; He et al. 2018 Zhang et al. 2020; Xiao et al. 2022;Mašek et al. 2018

^c^EDIC 2024; Azuara et al. 2013;Liang et al. 2018

^d^*bdl* below detection limit

#### Energy modelling of biochar utilisation scenario

During pyrolysis, the feedstock was heated from ambient temperature (${T}_{in}$, assumed to be 10 °C) to the reaction temperature (${T}_{py}$) of 600 °C. The heating requirement includes energy to heat the dry matter (${Heat}_{DM}$) and to vaporise the moisture content (${Heat}_{va}$) of the feedstock. A heat loss (${ef}_{loss}$) of 5% was assumed for the reactor, and the energy efficiency of the pyrolysis process (${ef}_{py}$) was assumed to be 50%. The heat required for straw pyrolysis was calculated using Eqs. 1–3.

1 $${Heat}_{py}^{straw}={(Heat}_{DM}+{Heat}_{va})/(1-{ef}_{loss})/ {ef}_{py}$$

2 $${Heat}_{DM}= {C}_{DM}*\left({T}_{py}-{T}_{in}\right)*{M}_{DM}$$

3 $${Heat}_{va}=\left({C}_{wa}*\left({T}_{va}-{T}_{in}\right)+{enth}_{va}+{C}_{va}*\left({T}_{py}-{T}_{va}\right)\right)*{M}_{mois}$$

where ${C}_{DM}$ is the heat capacity of the DM for the feedstock (1.61 kJ/kg·K for straw) (Ding and Jiang 2013), and ${M}_{DM}$ is the mass of the feedstock DM. ${C}_{wa}$ represents the heat capacity of water, which is 4.18 kJ/kg·K. ${T}_{va}$ is the boiling point of water, set at 373 K. ${enth}_{va}$ is the latent heat of vaporisation (2260 kJ/kg). ${C}_{va}$ is the heat capacity of water vapour, valued at 2.260 kJ/kg·K. ${M}_{mois}$ represents the mass of moisture in the feedstock.

Manure was processed through a dewatering process is used to reduce its high moisture content and improve energy efficiency. The process involved a decanter centrifuge followed by a roller press. The centrifuge separated slurry into a liquid fraction (2.02% DM) and a solid fraction (25.4% DM) (Pantelopoulos and Aronsson 2021). The solid was mixed with FYM and then processed through a roller press, resulting in a thick fraction with 33.3% DM and a thin fraction with 5.43% DM (Fournel et al. 2019). Following the full dewatering process, 702 kg of the thick fraction was recovered per t of FYM, and 61 kg per t of slurry. The remainder was directed to storage and land application.

The thick fraction was dried to 10% moisture content using a rotary drum dryer. The drying energy requirement was calculated by assuming that the thick fraction was heated from ambient temperature to the drying temperature (${T}_{dry}$) of 150 °C. The energy efficiency of the drying process (${ef}_{loss}$) was assumed to be 85% (Poels et al. 1987). The heat required for drying (${Heat}_{dry}$) was calculated using Eq. 4.

4 $${Heat}_{dry}={(C}_{DM}^{manure}*\left({T}_{dry}-{T}_{in}\right)*{M}_{DM}+\left({C}_{wa}*\left({T}_{va}-{T}_{in}\right)+{enth}_{va}+{C}_{va}*\left({T}_{dry}-{T}_{va}\right)\right)*\Delta {M}_{mois})/(1-{ef}_{loss})/ {ef}_{py}$$

where ${C}_{DM}^{manure}$ is the heat capacity of the dry matter in the thick fraction, with a value of 1.2 kJ/kg·K (Liu et al. 2014). $\Delta {M}_{mois}$ represents the reduction in moisture content before and after drying.

The dried thick fraction was then pyrolyzed. The heat required for manure pyrolysis (${Heat}_{py}^{manure}$) was calculated using Eqs. 5–6.

5 $${Heat}_{py}^{manure}={(Heat}_{DM}+{Heat}_{va}+{Heat}_{re})/(1-{ef}_{loss})/ {ef}_{py}$$

6 $${Heat}_{re}= {Q}_{py}*{M}_{DM}$$

where ${Heat}_{re}$ represents the heat required for the pyrolysis reaction of manure, and ${Q}_{py}$​ is the reaction heat demand of manure pyrolysis, which is 300 kJ/kg (Ro 2016).

**Fig. 3:**
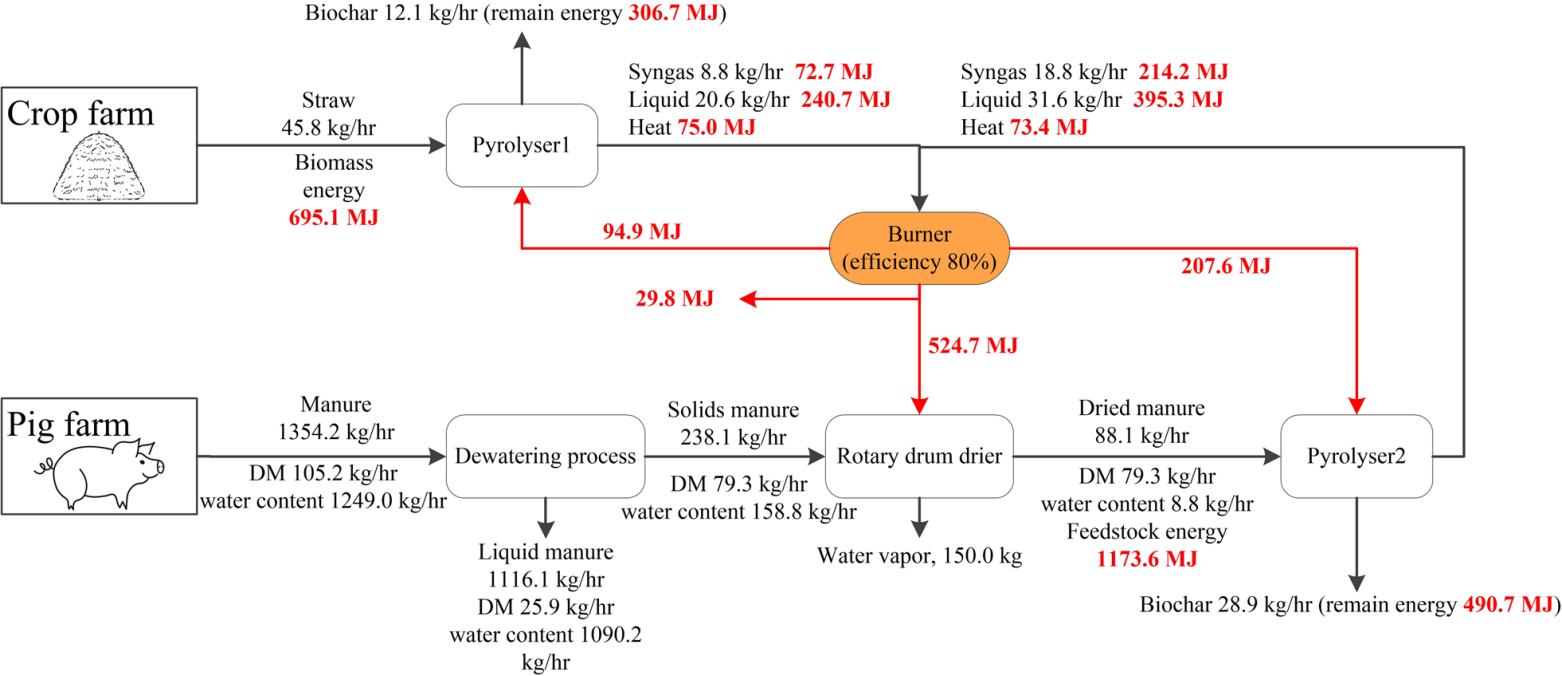
Energy and mass balance of the RB scenario

Syngas and liquid products from both pyrolysis lines were combusted to supply heat for pyrolysis and drying processes. For straw pyrolysis, HHVs for liquid and syngas were assumed to be 11.68 and 8.26 MJ/kg (Sedmihradská et al. 2020), respectively. In manure pyrolysis, the liquid phase comprised 52.6% organic and 47.4% aqueous content. The HHV of the organic phase was 27.1 MJ/kg, and that of syngas was 11.4 MJ/kg (Azuara et al. 2013). The burner was assumed to operate at a combustion efficiency of 80%. The energy and mass balance results are presented in Fig. 3.

#### Background data of facilities operation

All transport activities were assumed to occur within the farm, with a default transport distance of 1 km. Electricity was supplied from the national grid, and water is assumed to be tap water.

The material consumption data of biochar production system were derived from the BST-50 pyrolysis model (BESTON 2024), adjusted using exponential regression based on several continuous pyrolysis systems. A detailed inventory table is provided in Supplementary Table S1. The BST-50 is a continuous commercial-scale pyrolysis plant, operating at 600°C. The nominal feed capacity is between 10 and 15 m^3^/h, and the system operates with a water-based recycling cooling system. Given that the production scale in this study was significantly smaller, exponential regression (Eq. 7) was used to scale energy and material consumption. The baseline data for this regression were derived from multiple continuous pyrolysis systems (BESTON 2025). Then, the electricity and water consumption were proportionally scaled based on the scaling ratio ($F$) between the system and the BST-50 equipment.

7 $$F=26.09*\frac{{e}^{0.2972size}}{116}$$

The energy consumption of the centrifuge was estimated at 1 kWh/m^3^ of slurry (Szepessy and Thorwid 2018), and the roller press was assumed to consume 0.1 kWh/m^3^ of manure (Fournel et al. 2019). For drying, the energy consumption was based on an average of six studies, with a value of 2.85 kWh per 100 kg of dried manure (Poels et al. 1987).

### Life cycle impact assessment methodology

To evaluate the environmental implications of the proposed system in comparison with current practices, this study applied the 100-year global warming potential (GWP100) to quantify GHG emissions based on the LCI data. GWP100 is one of the most widely adopted metrics for assessing climate change impacts in LCA studies (Vallero 2019).

The life cycle impact assessment (LCIA) focused on three key elements: direct GHG emissions from the system, carbon sequestration through biochar application, and avoided emissions resulting from surplus heat recovery. Straw and manure are considered existing agricultural by-products; therefore, emissions associated with their production are excluded. In the RF scenario, the agricultural use of surplus straw is considered part of the short-term biogenic carbon cycle, and its associated CO₂ emissions are therefore excluded from the LCIA (IPCC 2022). As both feedstocks are derived from biological sources, CO_2_ emissions released during pyrolysis are considered biogenic and are not included in the system’s GHG accounting (Wang et al. 2020). It was assumed that biochar provides fertiliser benefits comparable to those of manure, and therefore the potential substitution of chemical fertilisers is not considered in this analysis (Liao et al. 2020).

GHG emissions from electricity consumption were calculated using the 2024 average grid emission factor for Great Britain (GB) (DESNZ and BEIS 2024). To examine the regional variation in environmental outcomes, spatially resolved electricity carbon intensity data were obtained from the National Energy System Operator (NESO 2025).These data offer half-hourly emission factors, from which annual average values were calculated for different GB regions.

The carbon sequestration potential of biochar was determined based on the proportion of stable carbon retained in soil over 100 years. As shown in Table 2, straw-derived biochar has a hydrogen-to-carbon molar (H:C) ratio below 0.4, corresponding to an estimated stability of 70%. Manure-derived biochar has an H:C ratio of 0.41, associated with 60% long-term stability, based on the recent research (Budai et al. 2013).

Manure management is a major source of CH_4_ and N_2_O emissions. The global warming potentials (GWPs) used to convert these gases to CO_2_ equivalents are 27.2 for CH_4_ and 273 for N_2_O (IPCC 2023).

The surplus heat generated by the biochar system can meet various on-farm thermal energy needs, including greenhouses and polytunnels heating, arable crops drying, and livestock production. The avoided emissions from surplus heat recovery (Kavindi et al. 2025) were calculated using the GWP100 factor associated with the “Heat, district or industrial, other than natural gas | market group for | Conseq, S” process in the Ecoinvent database.

### Techno-economic analysis methodology

For the TEA, the RF scenario represented the baseline and reflected the current farm operation, where only the transport costs associated with manure application are considered. In contrast, the RB represented the optimised system, and all potential costs associated with biochar production were accounted for. These included annualised capital expenditure (CAPEX), operating expenditure (OPEX), and the costs of feedstock handling and energy consumption.

The TEA methodology followed the guidelines that outlined in *Perry's Chemical Engineers' Handbook* (Perry and Green 2008). The cost of the pyrolysis equipment was based on a reference system with a processing capacity of 3 t per hour (BESTON 2024). The centrifuge cost was based on the Alfa Laval Aldec 45 model under full-capacity operation (Alfa Laval 2024). The roller press cost was derived from the equipment rated at 8 m^3^/h (HuberSE 2024), while the cost of the rotary drum dryer was based on an 11-t-per-hour system (Henan Mingyuan 2024). A scaling factor of 0.6 was applied where appropriate to adjust equipment costs (Tribe and Alpine 1986), and a discount rate of 5% was used to estimate the annualised CAPEX.

The key cost assumptions are summarised in Table 4. The unit cost of electricity was assumed to be 25.3 pence/kWh (DESNZ 2024b), while the unit water cost was £1.71/m^3^ (YorkshireWater 2024). Transport costs, based on Phase 1 of the biochar demonstrator project, were assumed to be £0.22 per t·km. While the surplus heat contributes to avoided GHG emissions, its economic benefits is not included in the analysis, as the relatively low energy grade is assumed to yield limited monetary value. However, incorporating these benefits would likely improve the economic favourability of the system.

**Table 4:** Parameters considered in the TEA

Parameter	Value/Comment
Base year	2024 (Jun)
Chemical engineering plant cost index (CEPCI)	CEPCI = 798.6^a^
	GBP/USD = 1.27
Currency	GBP
Plant lifespan (year)	8
UK location factor	1.02
Capital cost	
Pyrolysis equipment purchase cost	424,931
Centrifuge equipment purchase cost	24,650
Roller press equipment purchase cost	35,976
Dryer purchase cost	16,850
Equipment purchase cost (C_e_)	75,907
Inside battery limits (ISBL)	ISBL = 3.2*C_e_
Outside battery limits (OSBL)	OSBL = 0.4*ISBL
Fixed capital cost (CAPEX)	CAPEX = 5.0*C_e_
Fixed operating cost	
Labour (OL)	1 operator
	Average annual pay for operator = £25,694
Supervision	25% OL
Direct Ovhd	45% OL&Superv
Maintenance	3% of CAPEX
General plant overhead	65% OL&Maint
Land	2% of (ISBL + OSBL)
Insurance	1% of (ISBL + OSBL)

^a^(CE 2024)

### Sensitivity analysis

A sensitivity analysis was conducted to identify opportunities for reducing the high costs and enhancing the environmental performance of optimised agricultural residues management. This analysis evaluated the effects of variations in equipment cost, electricity consumption, biochar yield, straw production, and the DM content of the thick fraction following dewatering. Environmental indicators assessed included the unit cost of biochar and life cycle GHG emission reduction. Each parameter was independently varied by 5% to assess its relative impact on the results (Tang et al. 2024).

## Results

### Environmental impact results

**Fig. 4:**
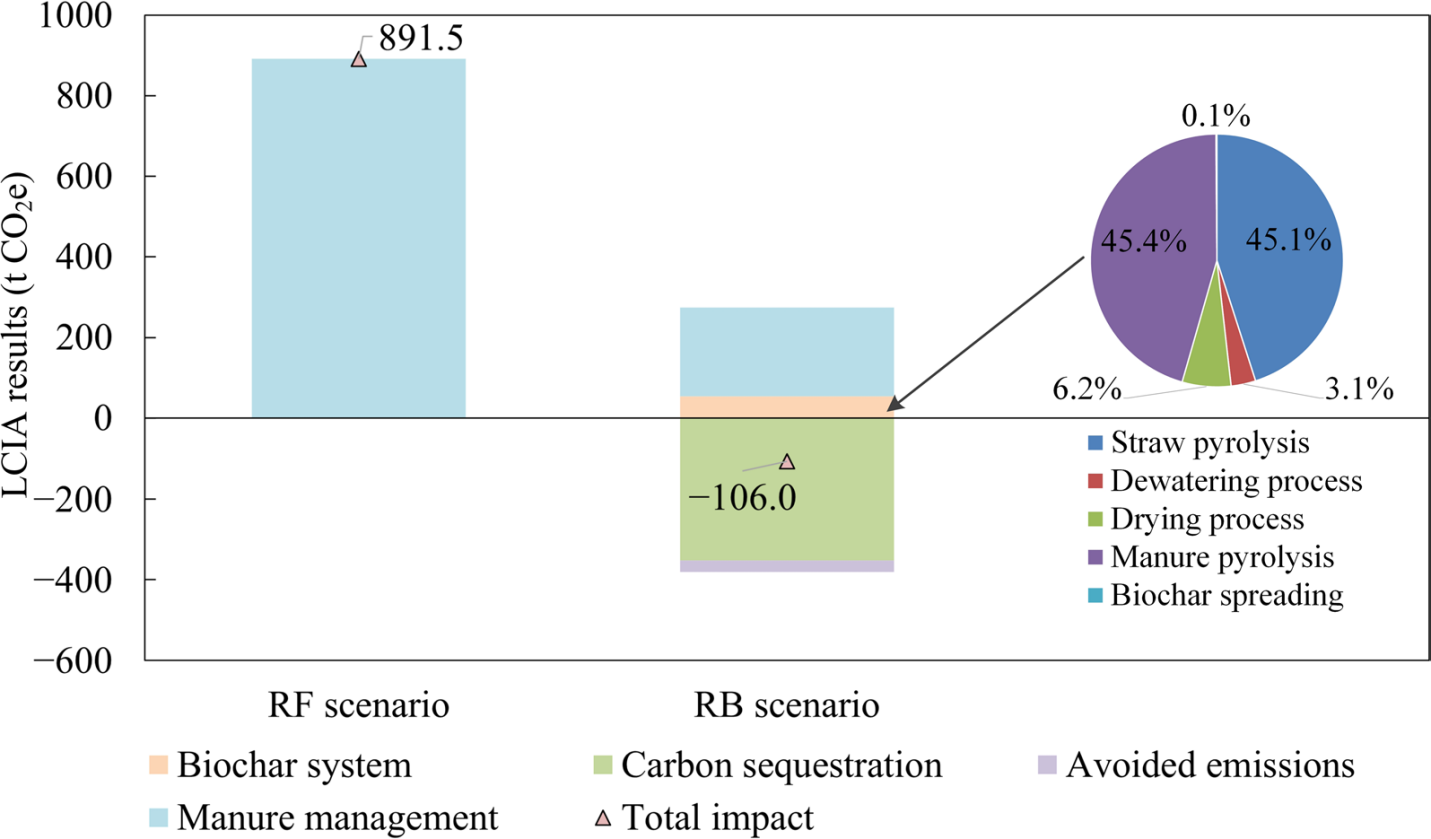
LCIA results of the proposed system compared with the reference scenario

The results of the life cycle GHG emission are presented in Fig. 4. The figure illustrates that the application of the proposed parallel biochar production system can achieve net negative GHG emission at the farm level. This substantial environmental benefit arose from three main contributing factors. Firstly, in the RB scenario, emissions were significantly reduced through the processing of 75% of the dry matter in manure, which mitigated emissions from both storage and land application. This resulted in an overall reduction of approximately 75% in manure management emissions. Secondly, biochar production enabled significant carbon sequestration, which accounted for 39% of the total emissions in the RF scenario. Thirdly, surplus heat generated by the system was used to meet the farm’s heat demand, contributing to an additional 29 t of avoided CO_2_e emissions.

Compared to the carbon sequestration from biochar, the emissions associated with the biochar system itself were relatively minor, amounting to 15% of the total sequestered carbon. These emissions were predominantly attributable to the pyrolysis of straw and manure, which together accounted for 90% of the system emissions. According to the LCI inputs, electricity consumption was the principal source of GHG emissions within the biochar system, representing 99.8% of total emissions.

### Economic impact results

**Fig. 5:**
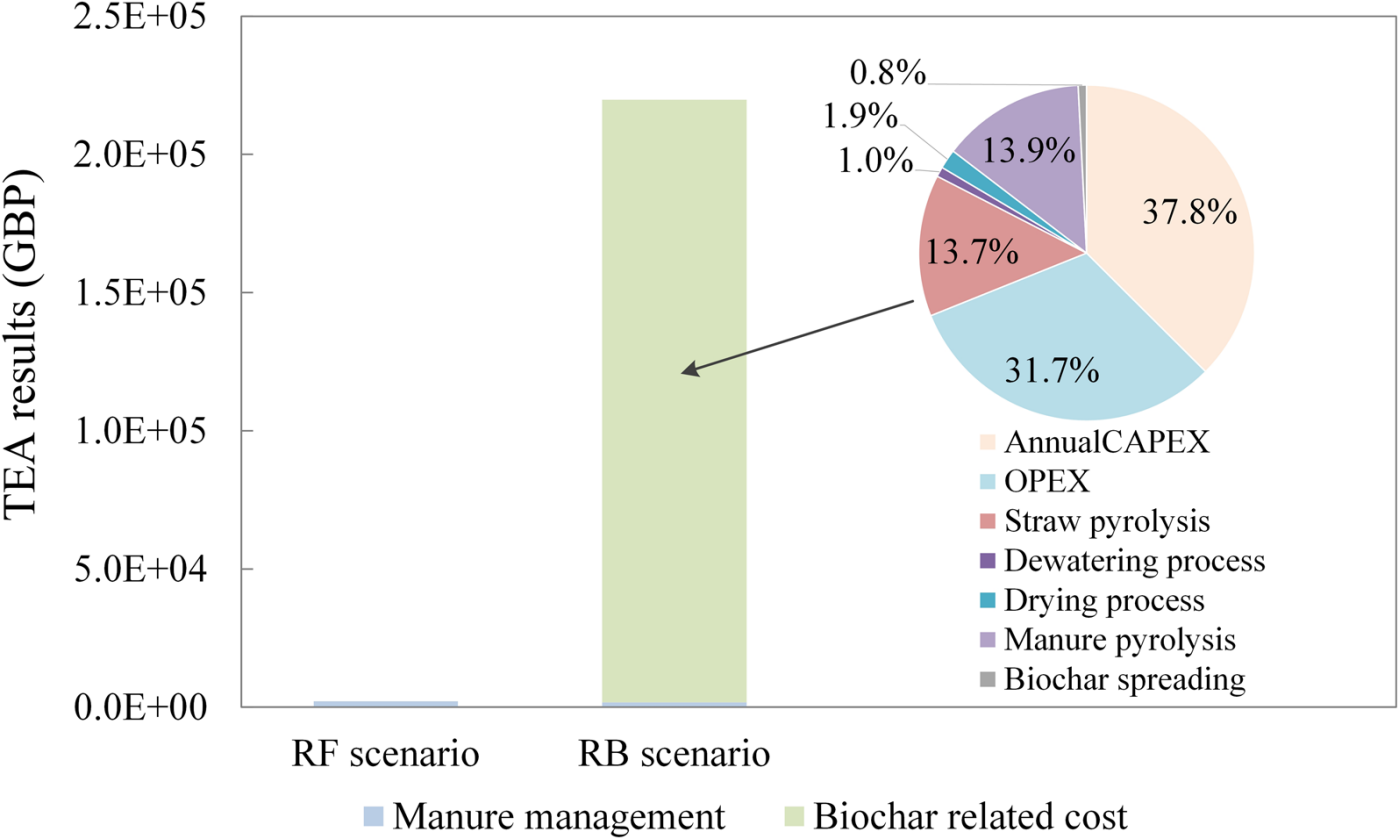
Techno-economic cost of the proposed system compared with the reference scenario

The TEA results (Fig. 5) indicated that, in contrast to the negligible cost associated with the RF scenario, the RB scenario required a substantial financial investment, amounting to £218,055 per year. When combined with the LCA results, the study found that compared to the RF scenario, the RB scenario achieved a reduction of 997.5 t CO_2_e in life cycle GHG emission at a cost of £225.6 per t. Additionally, it produced 295.5 t of biochar at a unit cost of £753.9 per t.

The largest contributor to the total cost of the biochar system was the annualised CAPEX, with the pyrolysis equipment, dewatering equipment, and dryer contributing 63%, 32%, and 5%, respectively. This was followed by the OPEX, over 90% of which was attributed to labour costs. Material and energy consumption costs related to the pyrolysis of straw and manure were the next major component, with electricity use representing 99% of this category. Other processes contributed minimally to the total costs. Overall, electricity consumption constituted approximately 30% of the total costs of the biochar system.

### Sensitivity analysis

A sensitivity analysis was conducted to evaluate the impact of the equipment cost, electricity consumption, biochar yield, straw production, and the DM content of the thick fraction after dewatering. The environmental indicators assessed were the unit cost of biochar and the cost of life cycle GHG emission reduction. Each parameter was varied independently by 5%.

**Fig. 6:**
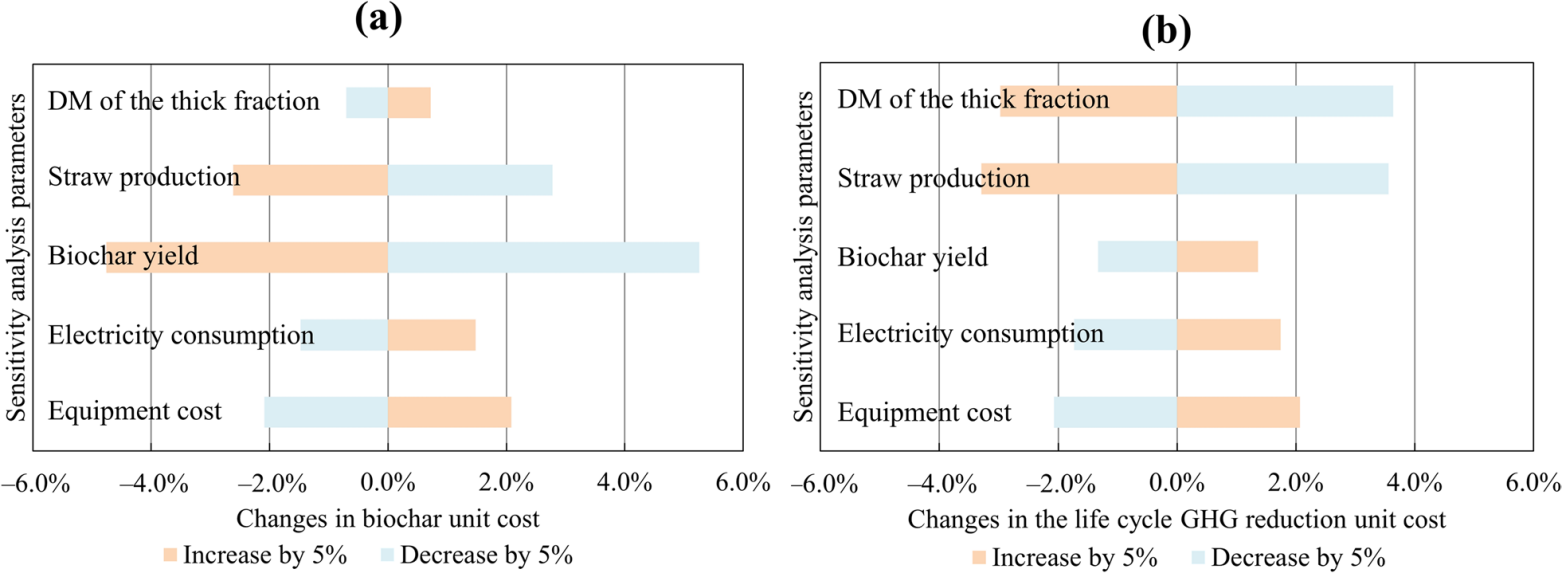
Sensitivity analysis results for: **a** biochar unit cost and **b** life cycle GHG emission reduction unit cost

Among all parameters, straw production has the greatest influence on both environmental indicators (Fig. 6). A 5% variation in straw production resulted in an approximate 3% change in the cost of biochar and a 3.5% change in the GHG abatement cost. Straw production is shaped by crop rotation and yield per hectare, both of which are subject to interannual variability. These dynamics are further explored in the following sections, using the farm’s 2021–2028 rotation plan and national yield statistics.

Biochar yield had significant effect on the unit cost of biochar, by around 5%, while its impact on the GHG abatement cost was smaller at 1.5%. A reduction in biochar yield increased the unit cost of biochar but decreased the unit cost of emissions reduction. This occurred because lower biochar yield led to greater surplus heat availability, which contributes more to avoided emissions.

The DM content of the thick fraction after dewatering affects the GHG abatement cost by approximately 3.3% but has minimal impact on the cost of carbon sequestration (0.7%). A decrease in DM content lowers the emissions reduction unit cost but raises the life cycle GHG reduction unit cost. A lower DM content increases the volume of manure processed, thereby enhancing emissions reduction from avoided manure management but raising electricity consumption, which in turn increased costs.

Changes in electricity consumption and equipment costs exerted a relatively moderate influence, with each parameter affecting both environmental indicators by approximately 1.5% and 2.1%, respectively.

### Impact of cropping rotation

**Fig. 7:**
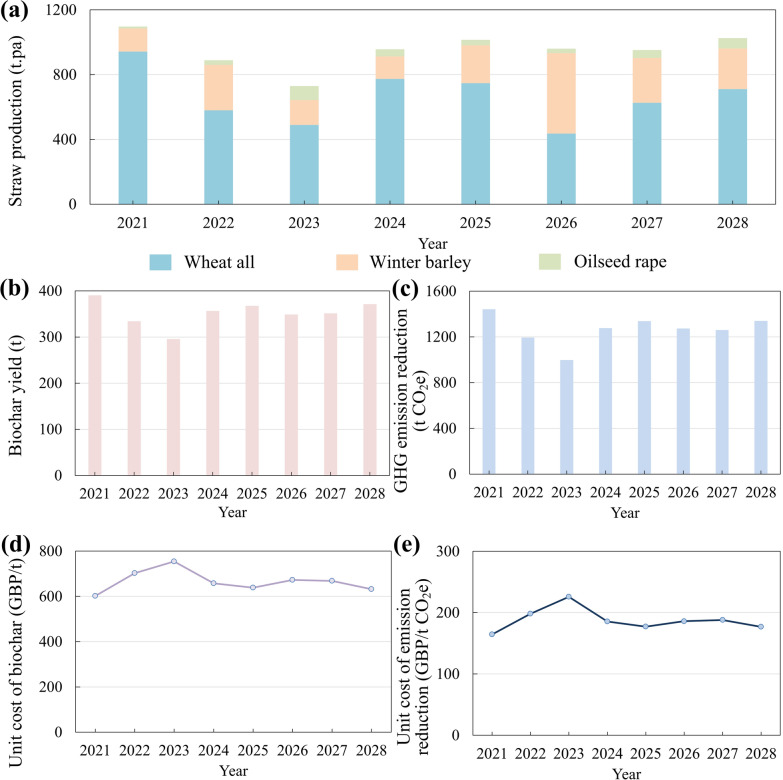
Impact of cropping rotation from 2021 to 2028 (estimated) for: **a** straw production, **b** biochar yield, **c** annual life cycle GHG emission reduction, **d** unit cost of biochar and **e** unit cost of life cycle GHG emission reduction

The sensitivity analysis revealed that fluctuations in straw production significantly affected the overall system performance. One of the primary factors contributing to this variability was crop rotation, which influences the consistency of straw availability and, consequently, affects both production costs and environmental outcomes. Based on the farm’s crop rotation schedule and yield data from 2023, straw production was estimated for the years 2021 to 2028 (Fig. 7a). The results revealed substantial interannual variation, with the highest production year 2021 yielding approximately one-third more straw than the lowest production year 2023 The proportion of different straw types also shiftsed, with wheat straw accounting for 86% of the total yield in 2021 but only 45% in 2026.

The study assumed that the biochar production equipment was sized to accommodate the maximum straw production over the eight-year rotation period. Unit costs of biochar and life cycle GHG emission reduction were calculated accordingly (Fig. 7b-e). The results showed that the environmental and economic performance of the system is strongly influenced by fluctuations in straw production driven by rotation. As straw production increased, the scale of biochar production expanded and the associated GHG mitigation improved. Correspondingly, the unit costs for both biochar production and emissions reduction declined with higher straw availability.

Although differences among straw types affected biochar characteristics, their influence on overall system performance was comparatively limited. Oilseed rape straw provided the highest biochar yield at 32.5% and the highest carbon content at 67.85%, suggesting strong carbon retention per unit of input. It also produced biochar with the highest energy content at 27.61 MJ/kg. However, its feedstock HHV was the lowest among the three at 12.04 MJ/kg, indicating limited potential for energy recovery from pyrolysis by-products. In contrast, wheat straw offered a more balanced profile, with a relatively high biochar yield and a higher feedstock HHV. These differences imply that oilseed rape straw may offer greater benefits for carbon sequestration, while wheat and barley straw may support more efficient energy recovery.

Overall, the system’s performance was primarily driven by the total straw availability rather than specific feedstock composition. Increasing production scale through greater straw production could substantially enhance both the environmental benefits and economic viability of the farm-level biochar system.

### Impact of straw yield per hectare

**Fig. 8:**
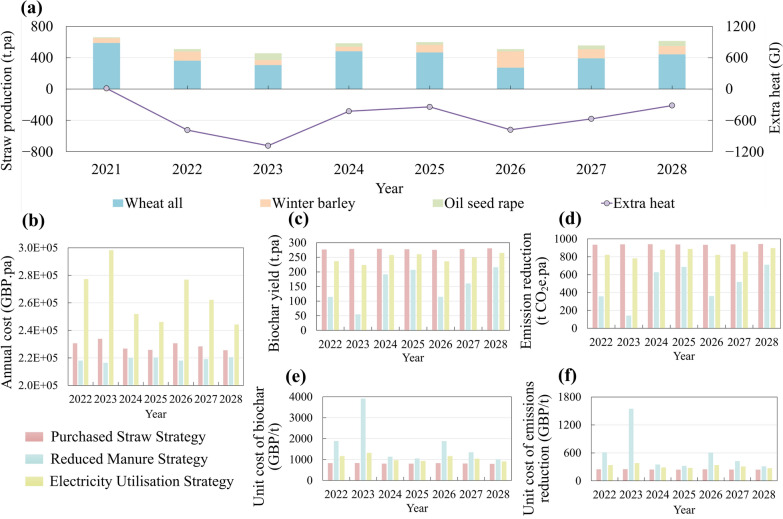
Environmental and economic impact of three strategies when straw is insufficient. **a** straw production, **b** annual cost of the biochar system, **c** biochar yield, **d** annual life cycle GHG emission reduction, **e** unit cost of biochar and **f** unit cost of life cycle GHG emission reduction

The proposed system utilised surplus heat generated from the straw-based pyrolysis process to meet the drying energy requirements of the manure-based line. However, this energy balance depended on the availability of straw. In addition to cropping rotation, straw yield per hectare is influenced by annual climatic factors such as solar radiation (Zhang et al. 2019), which affect the amount of surplus straw available. Although this study primarily relied on data from the University of Leeds farm for 2023, discussions with farm staff indicated that straw production varies considerably from year to year. To illustrate the potential impact of this variability, we simulated the system using national average straw yields, with government statistics reporting wheat and barley straw yields of 4 and 2.7 t per hectare (DEFRA 2024), respectively. Under these average conditions, the system failed to meet the manure line’s heat demand in five out of eight years (Fig. 8a), highlighting the risk of energy shortfalls under low straw-yield conditions.

To address this issue, we explored three operational strategies and assessed their environmental and economic impacts. The first strategy “PS” is the purchase of straw, where wheat straw is bought to meet the heat demand. The average market price of wheat straw (£56.7 per t) over the past five years was used for the TEA calculation(AHDB 2024) and 10 km was considered as the transportation distance. The second strategy “RM” is the reduction of manure, where the amount of manure treated is reduced in line with the available surplus heat from straw. The third strategy “EU” involves electricity utilisation, where electricity is consumed to supply the heat deficit. Taking 2023 as an example, if straw yield was based on national averages the resulting heat deficit was 1087 GJ. Under these conditions, the PS strategy would require the purchase of 207.5 t of wheat straw; and the RM strategy would limit manure processing to 19% of total volume; and EU strategy would consume 302,000 kWh of electricity.

Figure 8b-f presents the environmental and economic impacts of these strategies over an eight-year period. The PS strategy consistently delivered the highest environmental benefits at the lowest unit cost, producing 278 t of biochar and achieving an annual GHG reduction of 940 t CO_2_e. The RM strategy, while requiring no additional economic input, yielded the lowest environmental benefits and had the highest unit cost. Its effectiveness is highly sensitive to the scale of heat shortfall. In 2023, the year with the largest deficit, the unit cost of environmental benefits was more than six times higher than that of the PS strategy, while the emissions reductions achieved were just one-fifth. By contrast, in 2028, a year with minimal shortfall, the differences among the strategies were negligible. The EU strategy incurred the highest annual expenditure, with poorer environmental performance and higher costs compared to the PS strategy.

Overall, when straw production was insufficient to meet energy demands, purchasing straw to maintain energy balance emerged as the most effective approach to support farm-level decarbonisation. While reducing the volume of manure processed is the least costly option, it is the least favourable due to its limited environmental benefits and highest unit cost, particularly in years with substantial energy deficits.

## Discussion

### Life cycle interpretation

The LCIA and TEA results indicate that producing biochar from agricultural residues using the proposed parallel processing system can deliver substantial environmental benefits. For the case study farm, the system reduces GHG emissions from manure management by three quarters, enables carbon sequestration of 350 t with only 54 t of production-related emissions, and achieves 30 t of avoided emissions through heat substitution. However, these benefits come at a high cost, with a production cost of approximately £754 per t of biochar. This figure is consistent with estimates from previous studies (Table 5) but remains significantly higher than the current carbon price of around $90 per t CO_2_e (ICAP 2024), When the benefits from avoided emissions and improved manure management are included, the emission reduction cost decreases to £226 per t CO_2_e. Although this value is lower, it still exceeds the recommended cost of achieving negative emissions via biochar outlined in the UK's GGR policy report (BEIS 2019).

**Table 5:** Biochar production cost in recent research

Feedstock type	Cost (£/t)	Location	Year	Reference
Orchard waste	346–1422	United States	2021	Nematian et al. 2021
Chicken manure	1232	Korea	2015	Nguyen and Lee 2015
Lignocellulosic feedstocks	362–716	European Union	2020	Haeldermans et al. 2020
Sludge	596–967	United States	2020	Cheng et al. 2020
Poultry litter	167–218	UK	2015	Huang et al. 2015
This study	754	UK	2024	

Despite the current high costs, the TEA results highlight substantial opportunities to improve economic performance. Annualised CAPEX, OPEX, and energy consumption during the pyrolysis process each contribute roughly one-third to the total cost. Recent studies also indicate that biochar production costs are highly sensitive to these parameters (Shackley et al. 2011; Gamaralalage et al. 2025; Mari Selvam et al. 2024), suggesting that targeted optimisation in these areas could significantly enhance the system’s financial viability.

To make biochar technology economically viable compared to current carbon pricing, our analysis suggests that production costs would need to be reduced by approximately 70%. Such reductions are not unprecedented. For instance, the costs of solar photovoltaics (PV) and batteries have fallen by around 85% (Mandys et al. 2023) and 90% (IEA 2024), respectively, as a result of technological innovation and industry scale-up. These historical examples support the view that biochar technology, as it matures and scales, could achieve similar economic improvements through technological innovation and broader market adoption.

For small-scale on-farm biochar production, optimising reactor design (for example, through modular construction) and improving supply chain management for key equipment and materials are two promising approaches to cost reduction. Modular construction has been reported to lower infrastructure costs by approximately 15% compared to conventional designs (Mignacca and Locatelli 2020). Industry analyses indicate that supply chain optimisation can reduce costs by 5–10% (McKinsey & Company 2022).

Regarding OPEX, TEA analysis shows that 90% of these costs come from labour. This analysis assumes the employment of a dedicated operator. However, given the simplicity of small-scale pyrolysis equipment, biochar systems may be managed directly by trained farmers (Odesola and Owoseni 2010). If no additional labour is required, total system costs could be reduced by 29%. This would bring the cost of carbon abatement significantly closer to the current market benchmark, narrowing the cost gap by approximately 50%.

Electricity consumption accounts for 30% of the total production cost. In this study, electricity is assumed to be sourced entirely from the national grid. However, under the case study farm’s net zero plan, wind turbines and solar PV systems are expected to be deployed to supply electricity in the future (UoL 2022). This transition to renewables would reduce both the environmental and financial costs associated with grid-based electricity use.

Moreover, the small scale of the current system limits its energy efficiency. Expanding production capacity through cooperation among neighbouring farms could improve energy utilisation, reduce unit costs, and enhance overall feasibility (Tang et al. 2024). A community-based biochar system would allow farms to benefit from economies of scale while complying with feedstock-specific regulatory requirements and maintaining flexibility in residue management.

These findings highlight not only the current feasibility of biochar production at farm scale but also the potential for significant cost reductions through targeted optimisation. Future work may employ dynamic optimisation models to simulate the effects of specific technological upgrades, renewable integration, and operational improvements over time, further supporting the system’s long-term viability.

### Effect of the regional electricity carbon intensity

The environmental results indicate that electricity consumption is the dominant source of GHG emissions during the biochar production process, with the pyrolysis stage alone accounting for approximately 90% of total production-related emissions. This section evaluates how regional differences in electricity carbon intensity influence the life cycle GHG reduction achievable through the system.

**Fig. 9:**
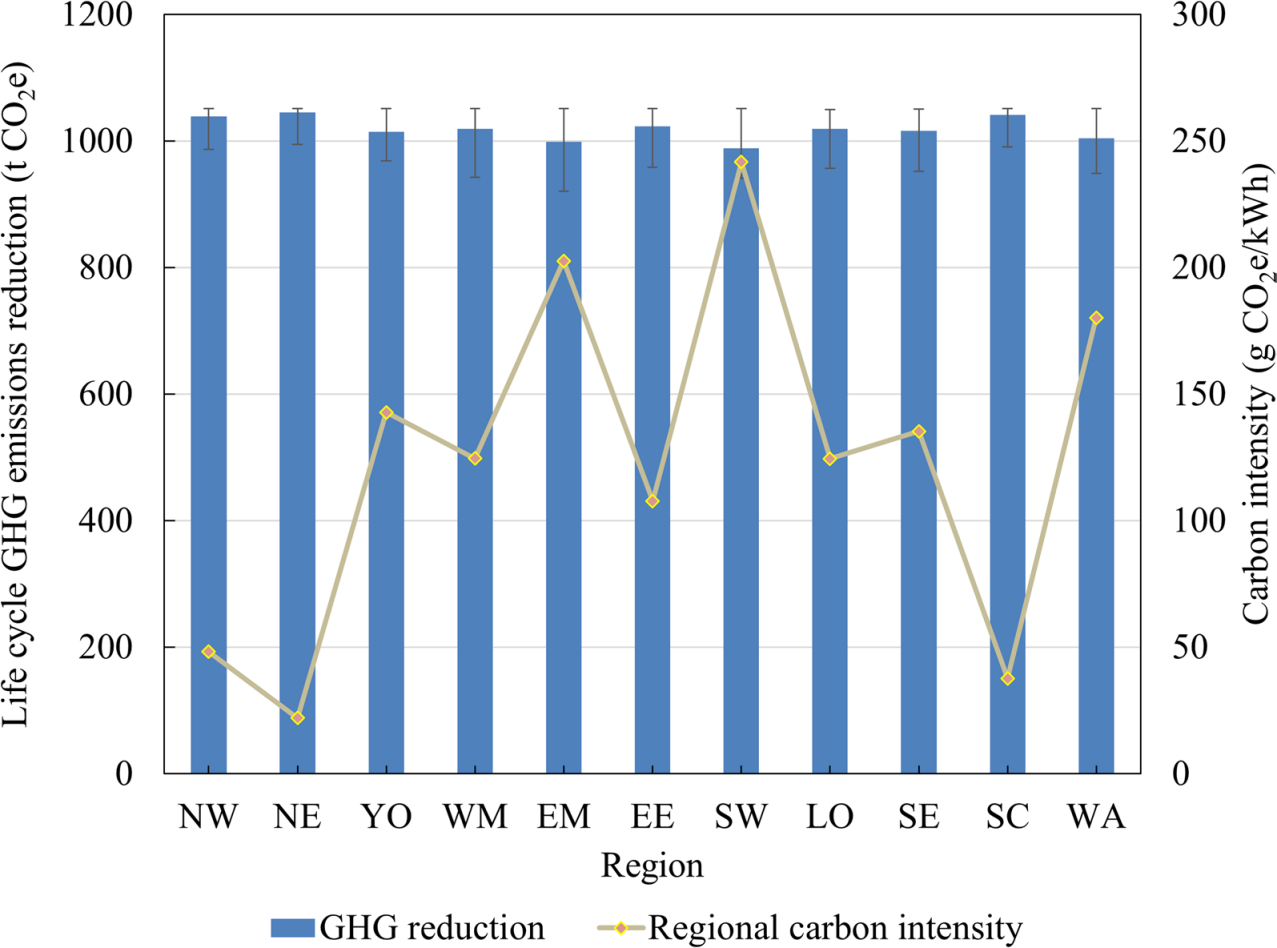
Life cycle GHG emissions reduction achieved by the farm-based integrated biochar production system in the different regions of GB. North East (NE), North West (NW), Yorkshire (YO), West Midlands (WM), East Midlands (EM), East of England (EE), South West (SW), London (LO), South East (SE), Scotland (SC) and Wales (WA)

Figure 9 illustrates that while regional variations in electricity carbon intensity introduce some differences, their overall impact on system performance is minor. All regions in GB achieve life cycle GHG emissions reduction of approximately 1000 t CO_2_e annually, demonstrating that the system is environmentally effective and applicable nationwide. Nonetheless, variations in carbon intensity across regions are still observable. For example, the South West, which has relatively low renewable energy penetration, exhibits a grid carbon intensity around 220gCO_2_e/kWh higher than that of the North East. As a result, assuming identical farm configurations and operating conditions, the total GHG reduction potential in the North East is approximately 5.6% higher than in the South West. Regions with higher shares of low-carbon electricity are better positioned to enhance the environmental performance of biochar systems. As the national grid decarbonises, the designed system is likely to deliver even greater climate benefits.

### Pathways to improve economic feasibility

Our analysis indicates that integrated biochar production from farm residues offers a promising solution for advancing GGR within the UK agricultural sector. The land application of biochar must comply with UK Environment Agency regulations, which prohibit mixing different feedstocks. The parallel production model proposed in this study addresses this challenge by enabling separate processing of multiple residue types, while simultaneously allowing for energy integration such as the reuse of surplus heat.

A key challenge for individual farms remains the economic feasibility of small-scale biochar production. High capital and operational costs (Hu et al. 2024; Campion et al. 2023), along with the complexity of processing (Meng et al. 2021), have limited the adoption of on-farm biochar systems. While most existing studies have focused on large-scale, centralised production benefiting from economies of scale and stable feedstock supply (Kochanek et al. 2022; Tang et al. 2024), such models are not readily applicable to distributed farm contexts. By demonstrating a practical, regulatory-compliant approach tailored to real-world farm constraints, our model highlights a feasible pathway for enabling biochar deployment at the small-farm scale.

However, system resilience may be compromised by annual fluctuations in straw availability caused by crop rotation and yield variability. Expanding the system from a single-farm operation to a cooperative model across multiple farms presents a potential solution. By pooling feedstock from neighbouring farms, the system can buffer against annual yield fluctuations and maintain stable production levels. Shared investment in larger-scale infrastructure may further improve energy efficiency and reduce per-unit costs, thereby enhancing the system’s economic performance. Additionally, larger-scale operations would produce more surplus heat, which could be repurposed to meet local energy demands, such as for greenhouse or heating purpose during winter months.

Although this study does not incorporate policy incentives in the economic assessment, their potential role should not be overlooked. For example, applying the UK Emissions Trading Scheme price of £90 per t CO_2_e could offset nearly 40% of the current carbon abatement cost. Similarly, biochar-specific subsidies or credits for sustainable residue management could help bridge the cost gap. Future research should explore how various market-based incentives might influence adoption decisions and improve the financial viability of biochar systems at both farm and community scales.

### Applicability across farm types and scales

The applicability of the parallel biochar production model depends on farm residue profiles, moisture management options, on-farm heat demand, access to supplementary residues, and practical constraints including labour and electricity sourcing. Residue composition and interannual variability shape both environmental and economic performance.

Arable-dominant farms, where straw supply is abundant, can operate the straw line at high utilisation and achieve lower unit costs. Farms with a balanced mix of straw and manure benefit from operational complementarity: the straw line supplies process heat for manure handling while separate processing maintains regulatory compliance. Livestock-dominant farms, where manure is prevalent and typically high in moisture, require careful sizing and pre-treatment to manage energy demand. In these contexts, importing straw to feed the heat line is the more economically favourable option, although overall costs remain higher than in straw-rich settings.

Scale is a further determinant of applicability. Small installations carry higher specific capital expenditure and higher unit carbon abatement costs. Increasing scale at a single site or forming a cooperative cluster improves capacity utilisation, enhances energy efficiency and reduces unit costs. Pooling residues also buffers the interannual variability associated with crop rotation and yield fluctuations, strengthening system resilience and improving the reliability of surplus heat for local uses.

In practice, aligning reactor sizing, pre-treatment, and heat integration with the local residue profile and heat demand enables the model to be adapted across farm types and scales. Where residues are stable and a suitable heat sink exists, a single-farm installation can be viable. Where residues are volatile or insufficient for year-round operation, cooperative configurations are preferable.

## Conclusion

This study developed and evaluated an integrated biochar production system using data from the University of Leeds Research Farm. The system introduces a novel parallel production model that separately processes straw and manure to comply with regulatory constraints, while enabling crossline heat recovery and integration. This represents a practical and adaptable solution for farm-scale biochar deployment, particularly in contexts with diverse agricultural residues.

Our findings demonstrate that even on a small-scale farm of 230 hectares, the proposed system can produce approximately 300 t of biochar per year, leading to a reduction of about 1000 t CO_2_e emissions annually. Emissions from manure management are reduced by 75%, and an additional 30 t CO_2_e are avoided through surplus heat utilisation.

However, these environmental benefits are associated with relatively high costs. The carbon abatement cost is estimated at £226 per t CO_2_e, with major contributions from annualised CAPEX (38%), OPEX (32%) and electricity consumption (30%). Although these figures are on the higher end of the ranges reported in previous studies, they nevertheless demonstrate that biochar systems can make a meaningful contribution to climate change mitigation at the farm level.

Sensitivity analysis identifies straw production as the dominant driver of performance. A 5% change in straw production leads to approximately a 3% change in the unit abatement cost. In low-yield years, heat shortfalls may constrain manure processing. Under such conditions, supplementing straw from external sources is more effective than reducing manure throughput or relying on additional electricity for drying. This highlights the value of multi-farm, community-based biochar systems for.

Overall, the proposed parallel production model advances existing approaches. It preserves regulatory compliance by processing straw and manure in dedicated lines while enabling heat integration across lines, which reduces the energy penalty of manure processing and improves capacity utilisation under farm conditions. Coupling the design with analysis at the farm scale for LCA and TEA provides configuration guidance by farm type and scale, and the modular architecture can scale from single-farm installations to cooperative hubs to raise capacity factors, lower unit costs and increase resilience to interannual residue variability.

This study has several limitations that warrant further investigation. It assumes agronomic equivalence between biochar and manure, is based on data from a single farm context, and excludes potential impacts of alternative straw management practices such as burning or ploughing. Future work should aim to validate these assumptions and expand the system boundary to enhance the generalisability and robustness of the findings.

## Supplementary Information

Supplementary material 1

## Data Availability

The data supporting this article is available on reasonable request from the authors.

## Declarations

### Competing of interests

There are no conflicts to declare.
